# Targeting Nrf2-Mediated Oxidative Stress Response in Traumatic Brain Injury: Therapeutic Perspectives of Phytochemicals

**DOI:** 10.1155/2022/1015791

**Published:** 2022-04-04

**Authors:** An-Guo Wu, Yuan-Yuan Yong, Yi-Ru Pan, Li Zhang, Jian-Ming Wu, Yue Zhang, Yong Tang, Jing Wei, Lu Yu, Betty Yuen-Kwan Law, Chong-Lin Yu, Jian Liu, Cai Lan, Ru-Xiang Xu, Xiao-Gang Zhou, Da-Lian Qin

**Affiliations:** ^1^Sichuan Key Medical Laboratory of New Drug Discovery and Drugability Evaluation, Materia Medica, Luzhou Key Laboratory of Activity Screening and Druggability Evaluation for Chinese Materia Medica, Education Ministry Key Laboratory of Medical Electrophysiology, School of Pharmacy, Southwest Medical University, Luzhou 646000, China; ^2^State Key Laboratory of Quality Research in Chinese Medicine, Macau University of Science and Technology, Taipa, Macau SAR, 99078, China; ^3^Department of Neurosurgery Sichuan Provincial People's Hospital, University of Electronic Science and Technology of China, Chengdu 610000, China

## Abstract

Traumatic brain injury (TBI), known as mechanical damage to the brain, impairs the normal function of the brain seriously. Its clinical symptoms manifest as behavioral impairment, cognitive decline, communication difficulties, etc. The pathophysiological mechanisms of TBI are complex and involve inflammatory response, oxidative stress, mitochondrial dysfunction, blood-brain barrier (BBB) disruption, and so on. Among them, oxidative stress, one of the important mechanisms, occurs at the beginning and accompanies the whole process of TBI. Most importantly, excessive oxidative stress causes BBB disruption and brings injury to lipids, proteins, and DNA, leading to the generation of lipid peroxidation, damage of nuclear and mitochondrial DNA, neuronal apoptosis, and neuroinflammatory response. Transcription factor NF-E2 related factor 2 (Nrf2), a basic leucine zipper protein, plays an important role in the regulation of antioxidant proteins, such as oxygenase-1(HO-1), NAD(P)H Quinone Dehydrogenase 1 (NQO1), and glutathione peroxidase (GPx), to protect against oxidative stress, neuroinflammation, and neuronal apoptosis. Recently, emerging evidence indicated the knockout (KO) of Nrf2 aggravates the pathology of TBI, while the treatment of Nrf2 activators inhibits neuronal apoptosis and neuroinflammatory responses via reducing oxidative damage. Phytochemicals from fruits, vegetables, grains, and other medical herbs have been demonstrated to activate the Nrf2 signaling pathway and exert neuroprotective effects in TBI. In this review, we emphasized the contributive role of oxidative stress in the pathology of TBI and the protective mechanism of the Nrf2-mediated oxidative stress response for the treatment of TBI. In addition, we summarized the research advances of phytochemicals, including polyphenols, terpenoids, natural pigments, and otherwise, in the activation of Nrf2 signaling and their potential therapies for TBI. Although there is still limited clinical application evidence for these natural Nrf2 activators, we believe that the combinational use of phytochemicals such as Nrf2 activators with gene and stem cell therapy will be a promising therapeutic strategy for TBI in the future.

## 1. Introduction

Traumatic brain injury (TBI) refers to the damage to the brain structure and function caused by mechanical and external forces, including two stages of primary injury and secondary injury [1]. It is a global neurological disease and is the biggest cause of death and disability in the population under 40 years of age [2]. The current clinical treatments for TBI mainly include interventional treatments such as hyperventilation, hypertonic therapy, hypothermia therapy, surgical treatment, drug therapy, hyperbaric oxygen therapy, and rehabilitation therapy [3, 4]. In the past few decades, the main interventions that have had the greatest impact and can reduce the mortality rate of severe TBI by several times are immediate surgical intervention and follow-up care by specialist intensive care physicians [5]. Post-traumatic intracranial hypertension (ICH) makes patient care more complicated, but new data shows that hypertonic therapy is the use of hypertonic solutions, such as mannitol and hypertonic saline (HTS) in the early treatment of ICH after severe TBI, which can reduce the burden of ICH and improve survival and functional outcomes [6]. Hypothermia therapy can reduce the effects of TBI through a variety of possible mechanisms, including reducing intracranial pressure (ICP), reducing innate inflammation, and brain metabolic rate. However, the results of a randomized POLAR clinical trial showed that early preventive hypothermia did not improve the neurological outcome at 6 months in patients with severe TBI [7]. Therefore, the effectiveness of hypothermia for TBI remains to be discussed. Surgical treatments include decompressive craniectomy (DC), which is a method that removes most of the top of the skull to reduce ICP and the subsequent harmful sequelae. However, the treatment effects for TBI are not satisfactory [8]. Many patients have a poor prognosis and will be left with serious disabilities and require lifelong care [9]. In addition, chemicals including corticosteroids, progesterone, erythropoietin, amantadine, tranexamic acid, citicoline, and recombinant interleukin-1 receptor (IL-1R) antagonist are used for the treatment of TBI [2]. However, these drugs are less safe and cannot work well, or may lead to unfavorable physiological conditions [10]. Recently, many studies have begun to investigate the possibility of using natural compounds with high safety as therapeutic interventions after TBI [11]. The latest evidence indicates that phytochemicals, including quercetin, curcumin, formononetin, and catechin, exert neuroprotective effects in TBI and other brain diseases via attenuating oxidative stress [12]. It is known to us, transcription factor NF-E2 related factor 2 (Nrf2) plays an important role in the regulation of heme oxygenase-1 (HO-1), NAD(P)H Quinone Dehydrogenase 1 (NQO1), glutathione peroxidase (GPx), and other antioxidant proteins, which ameliorates oxidative damage in TBI [13]. In this review, we discussed the critical role of oxidative stress in the pathology of TBI and the regulation of Nrf2-mediated oxidative stress response in TBI. In addition, we summarized the study advances of phytochemicals, including polyphenols, terpenoids, natural pigments, and otherwise, in the activation of Nrf2 signaling and their potential therapies for TBI in vivo and in vitro. Finally, we hope this review sheds light on the study on the treatment of TBI using phytochemicals as Nrf2 activators. Moreover, the combinational use of phytochemicals such as Nrf2 activators with gene and stem cell therapy will be a promising strategy for the treatment of TBI.

## 2. TBI

TBI, also known as acquired intracranial injury, occurs in the brain. It is caused by an external force, including a blow, bump, or jolt to the head, and the sudden and serious hit of the head by an object or the deep pierce of an object into the skull through the brain tissue [14]. According to the data from the Center for Disease Control and Prevention (CDC) of Unite States (U.S.), the most common causes mainly include violence, transportation, accidents, construction, and sports. In addition, there are about 288,000 hospitalizations for TBI a year, and males hold 78.8% [15, 16]. Usually, older adults (>75 years) have the highest rates of TBI. Therefore, TBI brings serious economic and spiritual burdens to the family and society [17].

**Figure 1:**
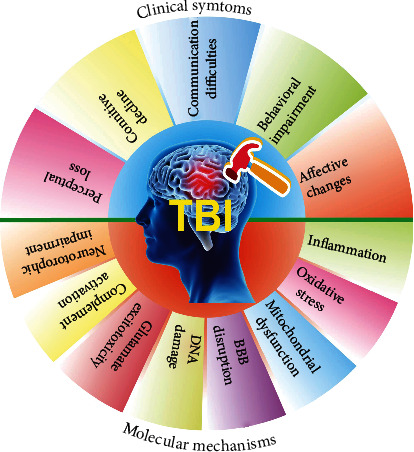
The clinical symptoms and molecular mechanism of TBI, an injury to the brain caused by an external force. The clinical symptoms of TBI mainly manifest as perceptual loss, cognitive decline, communication difficulties, behavioral impairment, and affective changes. The molecular mechanisms of TBI include inflammation, oxidative stress, mitochondrial dysfunction, blood-brain barrier (BBB) disruption, DNA damage, glutamate excitotoxicity, complement activation, and neurotrophic impairment.

TBI is classified in various ways, including type, severity, location, mechanism of injury, and the physiological response to injury [18]. In general, the Glasgow Coma Scale (GCS) score and neurobehavioral deficits are extensively used, and TBI is classified into mild, moderate, and severe types [19]. The clinical symptoms of TBI are greatly dependent on the severity of the brain injury and mainly include perceptual loss, cognitive decline, communication difficulties, behavioral impairment, affective changes, and otherwise [20] (Figure 1). The pathophysiology of TBI includes the primary injury, which is directly caused by physical forces, and the secondary injury referring to the further damage of tissue and cells in the brain [21]. The physical forces on the brain cause both focal and diffuse injuries. Emerging evidence indicates that patients who suffer from moderate or severe TBI are found to have focal and diffuse injuries simultaneously [22]. Most seriously, secondary brain injury is followed owing to the occurrence of biochemical, cellular, and physiological events during the primary brain injury [23]. Mechanistic studies demonstrate that several factors, including inflammation, oxidative stress, mitochondrial dysfunction, BBB disruption, DNA damage, glutamate excitotoxicity, complement activation, and neurotrophic impairment, are involved in the pathology and progression of TBI [24] (Figure 1). Currently, there is a growing body of studies showing that increasingly abnormal proteins or molecules are biomarkers closely associated with TBI, which helps to better understand the mechanism of TBI [25]. For example, the level of early structural damage biomarkers, including S100B protein in cerebrospinal fluid or blood nerve glial acidic protein, ubiquitin carboxyl-terminal hydrolase L1 and Tau helps to determine whether the head scan is required after TBI [26].

At present, the therapeutic strategies for TBI include hyperbaric oxygen therapy (HBOT), hyperventilation and hypertonic therapy, noninvasive brain stimulation, drug therapy, and biological therapy [27]. Most importantly, the combinational use of novel biological reagents (genes and stem cells) and pharmacological intervention preparations can decrease the complications and mortality of TBI [28]. Stem cell therapy includes stem cells regeneration transplantation and induction of endogenous stem cells activation through pharmacological or environmental stimuli. Several studies have shown that some drugs can not only improve the survival rate of stem cells but also enhance their efficacy. For example, the intravenous injection of mesenchymal stem cells (MSCs) and atorvastatin or simvastatin 24 hours after TBI could improve the recovery of modified neurological severity score (mNSS) [29]. In addition, the administration of calpain inhibitors 30 minutes after TBI followed by the transplantation of MSCs 24 hours after TBI could reduce the proinflammatory cytokines around the lesions, increase the survival rate of MSCs, and improve mNSS [30]. Moreover, the pretreatment with minocycline for 24 hours could protect transplanted stem cells from ischemia-reperfusion injury by inducing Nrf2 nuclear translocation and increasing the expression of downstream proteins [31]. Therefore, the in-deep clarification of the mechanism of TBI and adopting targeted methods for precise intervention will help the recovery of post-traumatic neurological function and further prevent the occurrence and development of complications, and ultimately open up a new way for effective treatment of TBI [32].

## 3. The Role of Oxidative Stress in TBI

Oxidative stress occurs owing to an imbalance of free radicals and antioxidants in the body, which lead to cell and tissue damage [32]. Therefore, oxidative stress plays a critical role in the development of diseases. As is known to us, diet, lifestyle, and environmental factors such as pollution and radiation contribute to the induction of oxidative stress, resulting in the excessive generation of free radicals [33]. In general, free radicals, including superoxide, hydroxyl radicals, and nitric oxide radicals, are molecules with one or more unpaired electrons [34]. It is well known that oxidative stress is implicated in the pathogenesis of various diseases, such as atherosclerosis, hypertension, diabetes mellitus, ischemic disease, neurodegeneration, and other central nervous system (CNS) related diseases [35, 36]. During normal metabolic processes, although many free radicals are generated, the body's cells can produce antioxidants to neutralize these free radicals and maintain a balance between antioxidants and free radicals [37]. A large body of evidence indicates that the overgenerated free radicals attack biological molecules, such as lipids, proteins, and DNA, ultimately breaking this balance and resulting in long-term oxidative stress [38]. However, oxidative stress also plays a useful role in some cases, such as physiologic adaptation and the modulation of intracellular signal transduction [39]. Thus, a more accurate definition of oxidative stress may be a state in which the oxidation system exceeds the antioxidant system owing to the occurrence of imbalance between them. At present, the biomarkers of oxidative stress, which are used to evaluate the pathological conditions of diseases and the efficacy of drugs, are becoming popular and attract increasing interest [40]. For example, lipid peroxides, 4-hydroxynonenal (4-HNE), and malondialdehyde (MDA) are the indicators of oxidative damage to lipids [41]. Thymine glycol (TG) and 8-oxoguanine (8-oxoG) are the biomarkers of oxidative damage to DNA [42]. In addition, a variety of proteins and amino acids, including carbonyl protein, dehydrovaline, nitrotyrosine, and hydroxyleucine, are oxidized and generate several products that are recognized to be biomarkers of oxidative stress. Among them, lipid peroxide as one of the most important biomarkers was determined in clinical. Furthermore, oxidative stress plays a pivotal role in the regulation of signaling transduction, including the activation of protein kinases and transcription factors, which affect many biological processes such as apoptosis, inflammatory response, and cell differentiation [43]. For example, gene transcription factors include nuclear factor *κ*B (NF-*κ*B) and activator protein-1 (AP-1) sense oxidative stress via oxidation and reduction cycling [44]. In addition, the generation of active oxygen species leads to the activation of NF-*κ*B, resulting in proinflammatory responses in various diseases such as neurodegenerative diseases, spinal cord injury, and TBI [45]. Therefore, oxidative stress is one of the important mechanisms that has been implicated in the pathology of CNS-related diseases.

Although the initial brain insult of TBI is an acute and irreversible primary damage to the parenchyma, the ensuing secondary brain injury progressing slowly over months to years seriously affects the treatment and prognosis of TBI [46]. Therefore, therapeutic interventions during secondary brain injury are essential. To date, many hallmarks are exhibited during delayed secondary CNS damage, mainly including mitochondrial dysfunction, Wallerian degeneration of axons, excitotoxicity, oxidative stress, and eventually neuronal death and overactivation of glial cells [24]. Recently, emerging evidence indicates that oxidative stress plays an important role in the development and pathogenesis of TBI [46]. In general, oxidative stress is resulted from or is accompanied by other molecular mechanisms, such as mitochondrial dysfunction, activation of neuroexcitation pathways, and activated neutrophils [47]. Kontos HA et al. first reported that superoxide radicals are immediately increased in brain microvessels after injury in a fluid percussion TBI model, while the scavengers of oxygen radicals including superoxide dismutase (SOD) and catalase significantly decrease the level of superoxide radicals and partly reverse the injury of the brain [48]. During the beginning minutes or hours after brain injury, a large number of superoxide radicals are generated owing to the enzymatic reaction or autoxidation of biogenic amine neurotransmitters, arachidonic acid cascade, damaged mitochondria, and oxidized extravasated hemoglobin [49]. Soon afterwards, the microglia are overactivated and neutrophils and macrophages are infiltrated, which also contribute to the production of superoxide radicals [50, 51]. In addition, iron overload and its resultant generation of several hydroxyl radicals and lipid peroxidation induce oxidative stress and neuronal ferroptosis, which significantly aggravate the pathogenesis of TBI from the following aspects, such as cerebral blood flow, brain plasticity, and the promotion of immunosuppression [52]. In this review, we focused on the research advance of the role of oxidative stress in TBI. At neutral pH, the iron in plasma is bound to the transferrin protein in the form of Fe^3+^, which also can be sequestered intracellularly by ferritin, an iron storage protein. Thus, iron in the brain is maintained at a relatively low level under normal conditions. However, the value of pH is decreased in the brain of TBI, which is accompanied by the release of iron from both transferrin and ferritin. Then, the excessive levels of active iron catalyze the oxygen radical reaction and induce oxidative damage and ferroptosis [53]. Additionally, hemoglobin is the second source that catalyzes the active iron after the mechanical trauma of the brain [54]. Iron is released from hemoglobin owing to the stimulation of hydrogen peroxide (H_2_O_2_) or lipid hydroperoxides, and the level of iron can be further increased as the pH decreases to 6.5 or even below [55]. Therefore, targeting the inhibition of iron levels by iron chelators may be a promising strategy for the treatment of TBI. For example, deferoxamine (DFO), a potent chelator of iron, can attenuate iron-induced long-term neurotoxicity and improve the spatial learning and memory deficit of TBI rats [56]. Moreover, nitric oxide (NO) is involved in the cascade of injury triggered by TBI. The activity of nitric oxide synthase (NOS) contributing to the generation of NO is increased as the accumulation of Ca^2+^ in TBI secondary injury. Then, NO reacts with free radical superoxide to generate “reactive nitrogen species” peroxynitrite (PN) in the forms of 3-nitrotyrosine (3-NE) and 4-HNE, which are found in the ipsilateral cortex and hippocampus of TBI animal models [24]. For example, N(omega)-nitro-L-arginine methyl ester (L-NAME), a NO-synthase inhibitor, was reported to attenuate neurological impairment in TBI and reduce the formation of NE and the number of NE-positive neurons [14]. Therefore, targeting the inhibition of oxidative stress in the brain is a promising strategy for the treatment of TBI.

## 4. Nrf2 Signaling-Mediated Oxidative Stress Response

**Figure 2:**
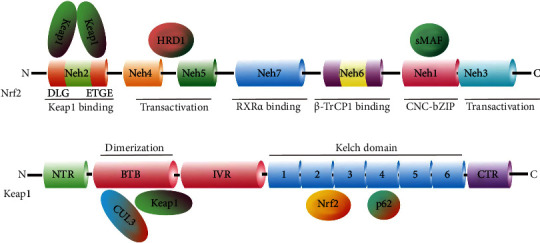
Structures of Nrf2 and Keap1 protein domains. (a) Nrf2 consists of 589 amino acids and has seven evolutionarily highly conserved domains (Neh1-7). Neh1 contains a bZIP motif and is responsible for DNA recognition and mediates the dimerization with the small MAF (sMAF) protein. Neh6 acts as a degron to mediate the degradation of Nrf2 in the nucleus. Neh4 and 5 are transactivation domains. Neh2 contains ETGE and DLG motifs which are required for the binding of Nrf2 to Keap1. Neh7 is a domain that interacts with RXR*α* to inhibit CNC-bZIP factors and the transcription of genes. Neh3 regulates CHD6. (b) Keap1 consists of 624 amino acids and has five domains. BTB domain together with the N-terminal region (NTR) of IVR to mediate the homodimerization of Keap1 and binding to Cul3. The Kelch domain and the C-terminal region (CTR) mediate the interaction with Neh2 of Nrf2 at the ETGE and DLG motifs.

In 1995, Itoh, K. et al. first discovered and reported that Nrf2 was the homolog of the hematopoietic transcription factor p45 NF-E2 [57]. To date, a total of 6 members including NF-E2, Nrf1, Nrf2, Nrf3, Bach1, and Bach2 are identified from the Cap “n” Collar (CNC) family [58]. Among them, Nrf2 is a conserved basic leucine zipper (bZIP) transcription factor. The literature reports that Nrf2 possesses seven highly conserved functional domains from NRF2-ECH homology 1 (Neh1) to Neh7, which are identified in multiple species including humans, mice, and chicken (Figure 2) [59]. Of these domains, Neh2, located in the N-terminal of Nrf2, possesses seven lysine residues and ETGE and DLG motifs, which are responsible for the ubiquitin conjugation and the binding of Nrf2 to its cytosolic repressor Keap1 at the Kelch domain, then facilitating the Cullin 3 (Cul3)-dependent E3 ubiquitination and proteasome degradation [60]. Both Neh4 and Neh5 domains with a lot of acidic residues act as transactivation domains to bind to cAMP response element-binding protein (CREB), which regulates the transactivation of Nrf2. Neh7 is a domain that interacts with the retinoic X receptor (RXR*α*), which can inhibit CNC-bZIP factors and the transcription of genes targeting Nrf2. Neh6 has two motifs including DSGIS and DSAPGS of *β*-transducing repeat-containing protein (*β*-TrCP) functioning as a substrate receptor for the Cul3-Rbx1/Roc1 ubiquitin ligase complex [61]. DSGIS is modulated by glycogen synthase kinase-3 (GSK-3) activity and enables *β*-TrCP to ubiquitinate Nrf2 [62]. The Neh1 domain has a Cap “N” Collar Basic Leucine Zipper (CNC-bZIP) DNA-binding motif, which allows Nrf2 to dimerize with small Maf proteins including MAFF, MAFG, and MAFK [63]. The Neh3 domain in the C-terminal of Nrf2 protein regulates chromoATPase/helicase DNA-binding protein 6 (CHD6), which is known as the Nrf2 transcriptional co-activator [64]. In addition, Neh3 also plays a role in the regulation of Nrf2 protein stability.

**Figure 3:**
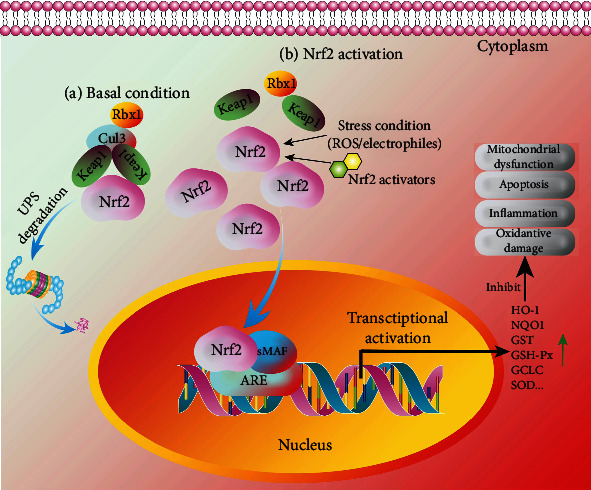
The regulation of the Nrf2 signaling pathway in TBI. Under basal conditions (a), Keap1 functions as a substrate adaptor protein for Cul3 to mediate the degradation of Nrf2 via the UPS pathway. Under Nrf2 activation (b), the stress condition or the treatment of Nrf2 activators induces the dissociation of Nrf2 from Keap1 and leads to the accumulation of Nrf2 in the cytoplasm and the nuclear translocation of Nrf2. Then, Nrf2 binds to sMAF and ARE to regulate the expression of its downstream transcription factors including HO-1, NQO1, GST, GSH-Px, GCLC, and SOD. Then, oxidative damage, inflammation, neuronal apoptosis, and mitochondrial dysfunction are inhibited.

Under normal conditions, Nrf2 is kept in the cytoplasm by a cluster of proteins including Keap1 and Cul3, which then undergoes degradation via the ubiquitin-proteasome system (UPS) [65]. In brief, Cul3 ubiquitinates Nrf2 and Keap1 act as a substrate adaptor to facilitate the reaction. Then, Nrf2 is transported to the proteasome for its degradation and recycling, and the half-time of Nrf2 is only 20 minutes. Under the condition of oxidative stress or the treatment of Nrf2 activators, the Keap1-Cul3 ubiquitination system is disrupted. Then, Nrf2 is translocated from the cytoplasm into the nucleus and forms a heterodimer with one of the sMAF proteins, which binds with the ARE and initiates the transcription of many antioxidative genes including HO-1, glutamate-cysteine ligase catalytic subunit (GCLC), SOD, and NQO1 (Figure 3). Emerging evidence indicates that Nrf2 is the most important protein that induces various gene expression to counter oxidative stress or activate the antioxidant response, which protects against cell damage, and death triggered by various stimuli including environmental factors such as pollution, lifestyle factors such as smoking or exercise, and other factors. There is a growing body of evidence showing that Nrf2 plays multiple roles in the regulation of oxidative stress, inflammation, metabolism, autophagy, mitochondrial physiology, and other biological processes [64]. It has been reported that Nrf2-KO mice are susceptible to suffering diseases, which are associated with oxidative damage [66]. Therefore, Nrf2 plays a critical role in cell defense and the regulation of cell survival in various diseases such as TBI.

## 5. The Potential Therapy of Phytochemicals as Nrf2 Activators in TBI

Because the primary injuries in TBI commonly result in acute physical damage and irreversible neuronal death, the therapies mainly aim at stabilizing the injury site and preventing it from secondary damage. As described above, the secondary damage of TBI is induced by various risks such as oxidative stress and develops progressively. To date, multiple therapeutic manners are developed, including the inhibition of excitotoxicity by glutamate receptor antagonists such as dexanbionol, the improvement of mitochondrial dysfunction using neuroprotective agents such as cyclosporine A, and the inhibition of axonal degeneration by calpain inhibitors such as MDL 28170 [67]. Emerging evidence indicates that oxidative stress is not only one of the pathogenesis of TBI but also the initiator and promoter of excitotoxicity, mitochondrial dysfunction, neuroinflammation, and other risks. Nrf2 plays a protective role in TBI via fighting against oxidative damage and inflammatory response in TBI [68], while the genetic deletion of Nrf2 delayed the recovery of post-TBI motor function and the cognitive function [69]. Therefore, targeting the discovery of Nrf2 activators to alleviate oxidative damage is a promising therapeutic strategy for TBI [70]. Recently, there are a lot of phytochemicals isolated from natural plants such as fruits, vegetables, grains, and other medicinal herbs and reported to activate the Nrf2 signaling pathway to exert neuroprotective effects in TBI [71]. In general, these natural phytochemicals as Nrf2 activators are used for the alleviation of the secondary damage of TBI. In this review, we summarize the research advances of phytochemicals, including polyphenols, terpenoids, natural pigments, and otherwise, in the activation of Nrf2 signaling and their potential therapies for TBI during secondary injury (Table 1).

### 5.1. Quercetin

Quercetin belonging to flavonoids is commonly found in dietary plants including vegetables and fruits such as onions, tomatoes, soy, and beans [72]. Emerging evidence indicates quercetin exerts a variety of pharmacological effects mainly involving antioxidation, anti-inflammation, antivirus, anticancer, neuroprotection, and cardiovascular protection [73]. It is known to us that the inflammatory response promotes oxidative damage in TBI [74]. In weight drop injury (WDI)-induced TBI mice, quercetin was reported to significantly inhibit neuroinflammation-medicated oxidative stress and histological alterations as demonstrated by the decreased lipid peroxidation and increased activities of SOD, catalase, and GPx [75]. Meanwhile, quercetin could significantly reduce the brain water content and improve the neurobehavioral status, which is closely associated with the activation of the Nrf2/HO-1 pathway [74]. The impairment of mitochondria function leads to an increase in reactive oxygen species (ROS) production and damages mitochondrial proteins, DNA, and lipids [72]. Quercetin was reported to significantly inhibit mitochondrial damage of TBI male Institute of Cancer Research (ICR) mice as evidenced by the decreased expression of Bax and increased levels of cytochrome c in mitochondria, as well as increased mitochondrial SOD and decreased mitochondrial MDA content, and the recovery of mitochondrial membrane potential (MMP) and intracellular ATP content. The mechanistic study demonstrated that quercetin promoted the translocation of Nrf2 from the cytoplasm to the nucleus, suggesting that quercetin exerts neuroprotective effects in TBI mice via maintaining mitochondrial homeostasis through the activation of Nrf2 signaling pathway [76]. In moderate TBI rats, quercetin inhibited oxidative nitrosative stress by reducing the activity of NOS including inducible nitric oxide synthase (iNOS) and constructive nitric oxide synthase (cNOS), as well as the concentration of thiobarbituric acid (TBA)-lipid peroxidation in the cerebral hemisphere and periodontal tissues [77]. Therefore, quercetin exerts neuroprotective effects in TBI via multiple biological activities, including inhibition of oxidative damage, nitrosative stress, and inflammatory response, as well as the improvement of mitochondrial dysfunction and neuronal function through the Nrf2 signaling pathway.

### 5.2. Curcumin

Curcumin, a polyphenol isolated from *Curcuma longa* rhizomes, has been reported to possess multiple biological activities, including antioxidative, anti-inflammatory, and anticancer effects [78]. Most importantly, curcumin is also demonstrated to cross the BBB and exert neuroprotection in various neurodegenerative diseases, such as Alzheimer's disease (AD), Parkinson's disease (PD), and amyotrophic lateral sclerosis (ALS), via the inhibition of neuronal death and neuroinflammation [79]. In addition, emerging evidence indicates that curcumin presents protective effects in TBI and activates the Nrf2 signaling pathway in vivo and in vitro [78, 80–82]. In mild fluid percussion injury (FPI)-induced TBI rats, curcumin significantly attenuated oxidative damage by decreasing the oxidized protein levels and reversing the reduction in the levels of brain-derived neurotrophic factor (DBNF), synapsin I, and cyclic AMP (cAMP)-response element-binding protein 1 (CREB) [81]. Meanwhile, curcumin improved the cognitive and behavioral function of TBI rats [81, 83–85]. In addition, the intraperitoneal administration of curcumin could improve the neurobehavioral function and decrease the brain water content in Feeney or Marmarou's weight drop-induced TBI mice. Furthermore, curcumin reduced the oxidative stress in the ipsilateral cortex by decreasing the level of MDA and increasing the levels of SOD and GPx, as well as promoted neuronal regeneration and inhibited neuronal apoptosis [80, 85]. Moreover, curcumin inhibited the neuroinflammatory response as demonstrated by the decreased number of myeloperoxidase (MPO) positive cells and increased levels of cytokines such as tumor necrosis factor-alpha (TNF-*α*), interleukin 6 (IL-6), and interleukin-1beta (IL-1*β*) [80]. The mechanistic study found that curcumin promoted the nuclear translocation of Nrf2 and increased the expression of downstream genes, including HO-1, NQO1, and GCLC, while the neuroprotective effects of curcumin including antioxidation, antiapoptosis, and anti-inflammation were attenuated in Nrf2-KO mice after TBI [80]. In addition, the anti-inflammatory effect of curcumin in TBI was also regulated by the TLR4/MyD88/NF-*κ*B signaling pathway [86] and aquaporin-4 (AQP4) [87]. Diffuse axonal injury (DAI), a type of TBI, is recognized as an important cause that results in long-term problems in motor and cognition, while curcumin could ameliorate axonal injury and neuronal degeneration of rats after DAI. In addition, curcumin overcame endoplasmic reticulum (ER) stress via strengthening the ability of the unfolded protein response (UPR) process and reducing the levels of plasma tau, *β*-APP, and NF-H. The mechanistic study revealed that curcumin activated the PERK/Nrf2 signaling pathway [88]. Most importantly, the combinational use of curcumin and candesartan, an angiotensin II receptor blocker used for the treatment of hypertension, showed better antioxidative, antiapoptotic, and anti-inflammatory effects than curcumin or candesartan alone [89]. In addition, tetrahydrocurcumin, the metabolite of curcumin, could also alleviate brain edema and reduce neuronal cell apoptosis, as well as improve neurobehavioral function via the Nrf2 signaling pathway in weight drop-induced TBI mice [90]. Taken together, curcumin together with its metabolites are useful for the treatment of TBI.

### 5.3. Formononetin

Formononetin, an O-methylated isoflavone phytoestrogen, is commonly found in plants such as red clover [91]. Accumulating studies show that formononetin has various biological activities, including the improvement of blood microcirculation, anticancer, and antioxidative [92]. In addition, formononetin exhibits neuroprotection in AD, PD, spinal cord injury, and TBI [93, 94]. It has been reported that the administration of formononetin could decrease the neurological score and cerebral moisture content of TBI rats [91]. In addition, the HE staining images showed that formononetin attenuated the edema and necrosis in the lesioned zones of the brain and increased the number of neural cells. At the same time, the oxidative stress was significantly reversed by formononetin as indicated by the increased enzymatic activity of SOD and GPx activity and decreased MDA content. The inflammatory cytokines including TNF-*α* and IL-6 as well as the mRNA level of Cyclooxygenase-2(COX-2) were also reduced by formononetin. The mechanistic study revealed that formononetin increased the protein expression of Nrf2 [95]. Furthermore, the same research team found that microRNA-155 (miR-155) is involved in the neuroprotection of formononetin in TBI. The pretreatment of formononetin significantly increased the expression of miR-155 and HO-1, which is accompanied by the downregulation of BACH1 [91]. All evidence suggests that formononetin provides neuroprotection in TBI via the Nrf2/HO-1 signaling pathway.

### 5.4. Baicalin

Baicalin, known as 7-D-Glucuronic acid-5,6-dihydroxyflavone, is a major flavone found in the radix of *Scutellaria baicalensis* [96]. Emerging evidence indicates that baicalin can cross the BBB and exert neuroprotective effects in various CNS-related diseases including AD, cerebral ischemia, spinal cord injury, and TBI [97]. In addition, baicalin was reported to activate the Nrf2 signaling pathway and attenuate subarachnoid hemorrhagic brain injury [98]. In weight drop-induced TBI mice, baicalin significantly reduced the neurological soft signs (NSS) score and the brain water content, and inhibited neuronal apoptosis as evidenced by the decreased terminal deoxynucleotidyl transferase dUTP nick end labeling (TUNEL)-positive neurons, Bax/Bcl-2 ratio, and the cleavage of caspase 3. Meanwhile, baicalin attenuated oxidative damage by decreasing MDA levels and increasing GPx and SOD activity and expression. The mechanistic study found that baicalin increased the expression of Nrf2 and promoted the nuclear translocation of Nrf2, meanwhile upregulated the mRNA and protein expression of HO-1 and NQO1, while the treatment of ly294002 reversed the effect of baicalin on antiapoptosis, antioxidation, and activation of the Nrf2 signaling pathway, suggesting that baicalin exerts neuroprotective effects via the Akt/Nrf2 pathway in TBI [96]. As is known to us, autophagy plays a protective mechanism in neurodegenerative diseases. Furthermore, the same research team found that baicalin induced autophagy and alleviated the BBB disruption and inhibited neuronal apoptosis of mice after TBI, while the co-treatment of 3-MA partly abolished the neuroprotective effect of baicalin. Therefore, baicalin provides a beneficial effect via the Nrf-2 regulated antioxidative pathway and autophagy induction.

### 5.5. Catechin

Catechin is a flavan-3-ol and belongs to a type of natural polyphenols [99]. It is a plant secondary metabolite and a potent antioxidant [100]. Structurally, it has four diastereoisomers, including two isomers with trans configuration called (+)-catechin and two isomers with cis configuration called (-)-epicatechin [101]. They are commonly found in food and fruits, such as cocoa, tea, and grapes. The pharmacological activity of catechin mainly involves antioxidative, anti-inflammatory, antifungal, antidiabetic, antibacterial, and antitumor effects [102]. In addition, catechin also exhibits neuroprotective effects in CCI-induced TBI rats by inhibiting the disruption of BBB and excessive inflammatory responses [103]. The expression of junction proteins including occludin and zonula occludens protein-1 (ZO-1) associated with BBB integrity was increased, while the levels of proinflammatory cytokines including IL-1*β*, iNOS, and IL-6 were decreased by catechin. At the same time, catechin significantly alleviated the brain damage as revealed by the decrease in the brain water content and brain infarction volume, as well as improved motor and cognitive deficits [103]. In addition, catechin inhibited cell apoptosis and induced neurotrophic factors in rats after TBI [104]. In CCI-induced TBI mice, the administration of epicatechin significantly attenuated the neutrophil infiltration and oxidative damage. Specifically, epicatechin could reduce lesion volume, edema, and cell death, as well as improve neurological function, cognitive performance, and depression-like behaviors. In addition, epicatechin decreased white matter injury, HO-1 expression, and the deposition of ferric iron. The mechanistic study found that epicatechin decreased the Keap1 expression while increasing the nuclear translocation of Nrf2. Meanwhile, epicatechin reduced the activity of Matrix metallopeptidase 9 (MMP9) and increased the expression of SOD1 and quinone 1 [102]. Therefore, epicatechin exerts neuroprotective effects in TBI mice via modulating Nrf2-regulated oxidative stress response and inhibiting iron deposition.

### 5.6. Fisetin

Fisetin, also known as 3,3′,4′,7-tetrahydroxyflavone, is a flavonol compound and was first extracted from *Cotinus coggygria* by Jacob Schmid in 1886 [105], and its structure was elucidated by Joseph Hergig in 1891. In addition, fisetin is also found in many vegetables and fruits, such as onions, cucumbers, persimmon, strawberries, and apples [106]. Emerging evidence indicates that fisetin acting as a potent antioxidant possesses multiple biological activities, including anti-inflammatory, antiviral, anticarcinogenic, and other effects [107]. Fisetin also presents neuroprotective effects via the antioxidative stress in AD, PD, etc. [108]. In addition, fisetin also showed protective effects in weight drop-induced TBI mice as shown by the decreased NSS, brain water content, Evans blue (EB) extravasation, and lesion volume of brain tissue, as well as the increased grip test score. Meanwhile, the MDA level was decreased and GPx activity was increased by fisetin, suggesting that fisetin provides a neuroprotective effect via suppressing TBI-induced oxidative stress [109]. In addition, the neuronal cell outline and structure stained by Nissl solution showed that fisetin improved neuronal viability, while neuronal apoptosis was inhibited by fisetin as demonstrated by the decreased TUNEL signals, and the reduced protein expression of Bax/Bcl-2 and cleaved caspase-3. The mechanistic study demonstrated that fisetin promoted the Nrf2 nuclear translocation and increased the expression of HO-1 and NQO1, while the KO of Nrf2 abrogated the neuroprotective effect of fisetin including antioxidation and antiapoptosis [109]. Moreover, fisetin was reported to exert anti-inflammatory effects in TBI mice via the TLR4/NF-*κ*B pathway, and the level of TNF-*α*, IL-1*β*, and IL-6 was significantly decreased. Meanwhile, the BBB disruption of TBI mice was attenuated by fisetin [110]. Therefore, fisetin exerts neuroprotective effects in TBI via the Nrf2-regulated oxidative stress and the NF-*κ*B-mediated inflammatory signaling pathway.

### 5.7. Luteolin

Luteolin, belonging to flavonoids, is abundant in fruits and vegetables such as carrots, green tea, and celery [111]. Emerging evidence indicates luteolin has a wide variety of biological activities including antioxidative and anti-inflammatory effects [112, 113]. In addition, several studies have demonstrated the neuroprotective effect of luteolin in multiple in vivo and in vitro models [114, 115]. For example, luteolin could recover motor performance and reduce post-traumatic cerebral edema in weight drop-induced TBI mice. The oxidative damage was reduced by luteolin as demonstrated by the decrease in MDA levels and the increase in GPx activity in the ipsilateral cortex. The mechanistic study found that luteolin promoted the nuclear translocation of Nrf2 and increased the mRNA and protein expressions of HO-1 and NQO1 [116]. In addition, luteolin significantly improved TBI-induced learning and memory impairment in rats after TBI, which was closely associated with the attenuation of oxidative damage indicated by the decreased MDA level and increased SOD and CAT activity [117, 118]. Therefore, the Nrf2-regulated oxidative stress response plays an important role in luteolin against TBI.

### 5.8. Isoliquiritigenin

Isoliquiritigenin, a chalcone compound, is often found in plants including *Sinofranchetia chinensis*, *Glycyrrhiza uralensis*, and *Dalbergia odorifera*. [119]. Isoliquiritigenin has been reported to attenuate oxidative damage, inhibit the inflammatory response, and suppress tumor growth [120]. In addition, isoliquiritigenin activates the Nrf2 signaling pathway to exert antioxidative and anti-inflammatory effects in multiple cellular and animal models. Isoliquirtigenin also exerts a neuroprotective effect in CCI-induced TBI mice via the Nrf2-ARE signaling pathway [121]. For example, isoliquiritigenin increased the Garcia Neuro score and decreased the brain water content, as well as the expression of aquaporin 4 (AQP4) and EB leakage. The glia activation indicated by GFAP expression was inhibited and the neuron viability showed by neurofilament light (NFL) expression was increased by isoliquiritigenin. In addition, isoliquiritigenin increased the number of Nissl staining-positive neurons and inhibited neuronal apoptosis as evidenced by the decreased expression of cleaved caspase-3. Furthermore, the oxidative damage was ameliorated by isoliquiritigenin as shown by the increased GPx activity, SOD levels, and decreased H_2_O_2_ concentration and MDA levels. However, the KO of Nrf2 significantly attenuated the neuroprotective effect of isoliquiritigenin in mice after TBI. The mechanistic study demonstrated that isoliquiritigenin increased the nuclear translocation of Nrf2 and the protein and mRNA expression of NQO1 and HO-1. In the in vitro study, isoliquiritigenin also activated the Nrf2-ARE signaling pathway and increased the cell viability in oxygen and glucose deprivation (OGD)-induced SH-SY5Y cells. In addition, isoliquiritigenin inhibited shear stress-induced cell apoptosis in SH-SY5Y cells, as well as suppressed the inflammatory response and inhibited neuronal apoptosis in CCI-induced TBI mice or rats via the PI3K/AKT/GSK-3*β*/NF-*κ*B signaling pathway [122, 123]. Moreover, isoliquiritigenin protected against BBB damage in mice after TBI via inhibiting the PI3K/AKT/GSK-3*β* pathway [123]. Therefore, isoliquiritigenin may be a promising agent for the treatment of TBI via the inhibition of oxidative stress, inflammatory response, and BBB disruption in TBI.

### 5.9. Tannic Acid

Tannic acid, a natural polyphenol, is commonly found in green and black teas as well as nuts, fruits, and vegetables [124]. Emerging evidence indicates that tannic acid possesses multiple biological activities such as antioxidative, anti-inflammatory, antiviral, and antiapoptotic effects [125–127]. In addition, tannic acid exhibits neuroprotective effects as shown by the improvement of behavioral deficits and the inhibition of neurodegeneration [128]. Recently, tannic acid has been proven to ameliorate the oxidative damage and behavioral impairments of mice after TBI [128]. For example, tannic acid significantly increased the score in the grip test and the motor coordination time as well as decreased the stay time in the balance test. In addition, tannic acid inhibited neuronal damage and reduced the brain water content of TBI mice. A further study found that tannic acid could attenuate oxidative stress as evidenced by increased glutathione (GSH) levels, 1-Chloro-2,4-dinitrobenzene (CDNB) conjunction, NADPH oxidation, and H_2_O_2_ consumption. In addition, apoptosis-related proteins including cleaved caspase-3 and Poly (ADP-ribose) polymerase (PARP), as well as Bax/Bcl-2, were significantly inhibited by tannic acid. Meanwhile, the inflammatory response indicated by the increased levels of TNF-*α* and IL-1*β* and GFAP immunofluorescence intensity was also suppressed. The mechanistic study demonstrated that tannic acid increased the protein expression of Nrf2, PGC-1*α*, Tfam, and HO-1. Therefore, tannic acid exerts a neuroprotective effect in TBI via activating the PGC-1*α*/Nrf2/HO-1 signaling pathway.

### 5.10. Ellagic Acid

Ellagic acid, an innate polyphenol, is commonly found in various berries such as blueberries, strawberries, blackberries, together with walnuts and nuts [129]. Several studies show that ellagic acid exerts multiple biological activities, including anti-inflammatory, antioxidative, antifibrosis, antidepressant, and neuroprotective effects [130]. In addition, ellagic acid also exhibits protective effects in various brain injuries such as neonatal hypoxic brain injury, cerebral ischemia/reperfusion injury, carbon tetrachloride (CCl4)-induced brain damage, and TBI [131–133]. Here, we summarize the neuroprotective effect of ellagic acid in TBI and its mechanism of action. In experimental diffuse TBI rats, the treatment of ellagic acid significantly improved memory and hippocampus electrophysiology deficits [134]. Meanwhile, the inflammatory responses indicated by the elevated TNF-*α*, IL-1*β*, and IL-6 levels were reduced by ellagic acid [134, 135]. In addition, ellagic acid could also decrease the BBB permeability of mice after TBI [135]. In CCl4-induced brain injury rats, ellagic acid decreased MDA levels, increased GSH content, and CAT activity. The mechanistic study demonstrated that ellagic acid inhibited the protein expression of NF-*κ*B and COX-2 while increasing the protein expression of Nrf2 [133]. Therefore, ellagic acid exerts an antioxidative effect via activating the Nrf2 pathway and exhibits anti-inflammatory effects via inhibiting the NF-*κ*B pathway in TBI.

### 5.11. Breviscapine

Breviscapine is an aglycone flavonoid and is isolated from the Erigeron plant [136]. Modern pharmacological studies indicate that breviscapine can expand blood vessels to assist in microcirculation, suggesting its potential therapeutic role in cardiovascular and CNS-related diseases [137]. In addition, breviscapine, acting as a scavenger of oxygen-free radicals, is demonstrated to improve ATPase and SOD activity. Recently, breviscapine is also reported to improve neurobehavior and decrease neuronal apoptosis in TBI mice, which is closely associated with the translocation of Nrf2 from the cytoplasm into the nuclear and the subsequent upregulation of Nrf2 downstream factors such as HO-1 and NQO1 [138]. In addition, the inhibition of glycogen synthase kinase-3*β* (GSK-3*β*) and IL-6 by breviscapine is associated with its neuroprotective effect in TBI [139, 140]. Therefore, breviscapine exerts neuroprotective effects in TBI via antioxidation, antiapoptosis, and anti-inflammatory responses.

### 5.12. Asiatic Acid

Asiatic acid belonging to pentacyclic triterpene is isolated from natural plants such as *Centella asiatica* [141]. Studies have shown that asiatic acid exhibits potent anti-inflammatory and antioxidative properties, which contributes to its protective effects in spinal cord injury, ischemic stroke, cardiac hypertrophy, liver injury, and lung injury through multiple mechanisms [142]. For example, the administration of asiatic acid could increase Basso, Beattie, Bresnahan scores and the plane test score in spinal cord injury (SCI) rats. Meanwhile, asiatic acid inhibited the inflammatory response by reducing the levels of IL-1*β*, IL-18, IL-6, and TNF-*α*, and counteracted oxidative stress by decreasing ROS, H_2_O_2_, and MDA levels while increasing SOD activity and glutathione production. The underlying mechanisms include the activation of Nrf2/HO-1 and the inhibition of the NLRP3 inflammasome pathway. In addition, asiatic acid could alleviate tert-butyl hydroperoxide (tBHP)-induced oxidative stress in HepG2 cells. The researchers found that asiatic acid significantly inhibited tBHP-induced cytotoxicity, apoptosis, and the generation of ROS, which attributed to the activation of Keap1/Nrf2/ARE signaling pathway and the upregulation of transcription factors including HO-1, NQO-1, and GCLC [143]. In a CCI-induced TBI model, the administration of asiatic acid significantly improved neurological deficits and reduced brain edema. Meanwhile, asiatic acid counteracted oxidative damage as evidenced by the reduced levels of MDA, 4-HNE, and 8-hydroxy-2′-deoxyguanosine (8-OHdG). The mechanistic study further found that asiatic acid could increase the mRNA and protein expression of Nrf2 and HO-1 [144]. Taken together, asiatic acid improves neurological deficits in TBI via activating the Nrf2/HO-1 signaling pathway.

### 5.13. Aucubin

Aucubin, an iridoid glycoside isolated from natural plants such as *Eucommia ulmoides* [145], is reported to have several pharmacological effects including antioxidation, antifibrosis, antiageing, and anti-inflammation [145–147]. Recently, emerging evidence indicates that aucubin exerts neuroprotective effects via antioxidation and anti-inflammation [148]. In addition, aucubin also inhibited lipid accumulation and attenuated oxidative stress via activating the Nrf2/HO-1 and AMP-activated protein kinase (AMPK) signaling pathways [147]. Moreover, aucubin inhibited lipopolysaccharide (LPS)-induced acute pulmonary injury through the regulation of Nrf2 and AMPK pathways [149]. In H_2_O_2_-induced primary cortical neurons and weight drop-induced TBI mouse model, aucubin was found to significantly decrease the excessive generation of ROS and inhibit neuronal apoptosis. In addition, aucubin could reduce brain edema, improve cognitive function, decrease neural apoptosis and loss of neurons, attenuate oxidative stress, and suppress the inflammatory response in the cortex of TBI mice. The mechanistic study demonstrated that aucubin activated the Nrf2-ARE signaling pathway and upregulated the expression of HO-1 and NQO1, while the neuroprotective effect of aucubin in Nrf2-KO mice after TBI was reversed [150]. Therefore, aucubin provides a protective effect in TBI via activating the Nrf2 signaling pathway.

### 5.14. Ursolic Acid

Ursolic acid, a pentacyclic triterpenoid compound, is widely found in various fruits and vegetables such as apples, bilberries, lavender, and hawthorn. [151]. Ursolic acid has been reported to possess multiple pharmacological effects including anti-inflammatory, antioxidative, antifungal, antibacterial, and neuroprotective properties [152]. In addition, ursolic acid activates the Nrf2/ARE signaling pathway to exert a protective effect in cerebral ischemia, liver fibrosis, and TBI [153]. In weight drop-induced TBI mice, the administration of ursolic acid could improve neurobehavioral functions and reduce the cerebral edema of mice after TBI. In addition, ursolic acid inhibited neuronal apoptosis as shown by the Nissl staining images and TUNEL staining. Meanwhile, ursolic acid ameliorated oxidative stress by increasing SOD and GPx activity as well as decreasing MDA levels. The mechanistic study demonstrated that ursolic acid promoted the nuclear translocation of Nrf2 and increased the levels of transcription factors including HO-1 and NQO1, while the KO of Nrf2 could partly abolish the protective effect of ursolic acid in TBI [153]. Therefore, ursolic acid exerts a neuroprotective effect in TBI via partly activating the Nrf2 signaling pathway.

### 5.15. Carnosic Acid

Carnosic acid, a natural benzenediol abietane diterpene, is found in *Rosmarinus officinalis* and *Salvia officinalis* [154]. Carnosic acid and carnosol are two major antioxidants in *Rosmarinus officinalis* [155]. Emerging evidence indicates that carnosic acid is a potent activator of Nrf2 and exerts a neuroprotective effect in various neurodegenerative diseases [156]. In CCI-induced acute post-TBI mice, carnosic acid could reduce TBI-induced oxidative damage by decreasing the level of 4-HNE and 3-NE in the brain tissues. A further study demonstrated that carnosic acid maintained mitochondrial respiratory function and attenuated oxidative damage by reducing the amount of 4-HNE bound to cortical mitochondria [157, 158]. In addition, carnosic acid showed a potent neuroprotective effect in repetitive mild TBI (rmTBI) as evidenced by the significant improvement of motor and cognitive performance. Meanwhile, the expression of GFAP and Iba1 expression was inhibited, suggesting that carnosic acid inhibited the neuroinflammation in TBI [159]. Therefore, carnosic acid exerts a neuroprotective effect via inhibiting the mitochondrial oxidative damage in TBI through the Nrf2-ARE signaling pathway.

### 5.16. Fucoxanthin

Fucoxanthin, a carotenoid isolated from natural plants such as seaweeds and microalgae, is considered a potent antioxidant [160]. Several studies show that fucoxanthin exerts various pharmacological activities such as antioxidation, anti-inflammation, anticancer, and health protection effects [161]. In addition, fucoxanthin exerts anti-inflammatory effects in LPS-induced BV-2 microglial cells via increasing the Nrf2/HO-1 signaling pathway [162] and inhibits the overactivation of NLRP3 inflammasome via the NF-*κ*B signaling pathway in bone marrow-derived immune cells and astrocytes [163]. In mouse hepatic BNL CL.2 cells, fucoxanthin was reported to upregulate the mRNA and protein expression of HO-1 and NQO1 via increasing the phosphorylation of ERK and p38 and activating the Nrf2/ARE pathway, which contributes to its antioxidant activity [164]. Recently, it has been reported that the neuroprotective effect of fucoxanthin in TBI mice was regulated via the Nrf2-ARE and Nrf2-autophagy pathways [165]. In this study, the researchers found that fucoxanthin alleviated neurological deficits, cerebral edema, brain lesions, and neuronal apoptosis of TBI mice. In addition, fucoxanthin significantly decreased the generation of MDA and increased the activity of GPx, suggesting its antioxidative effect in TBI. Furthermore, in vitro experiments revealed that fucoxanthin could improve neuronal survival and reduce the production of ROS in primary cultured neurons. A further mechanistic study revealed that fucoxanthin activated the Nrf2-ARE pathway and autophagy in vivo and in vitro, while fucoxanthin failed to activate autophagy and exert a neuroprotective effect in Nrf2^−/−^ mice after TBI. Therefore, fucoxanthin activates the Nrf-2 signaling pathway and induces autophagy to exert a neuroprotective effect in TBI.

### 5.17. *β*-carotene

*β*-carotene, abundant in fungi, plants, and fruits, is a member of the carotenes and belongs to terpenoids [166]. Accumulating studies indicate that *β*-carotene acting as an antioxidant has potential therapeutic effects in various diseases, such as cardiovascular disease, cancer, and neurodegenerative diseases [167, 168]. Meanwhile, the neuroprotective effect of *β*-carotene was also reported in CCI-induced TBI mice; the administration of *β*-carotene significantly improved neurological function and brain edema as evidenced by the decreased neurological deficit score and brain water content and increased time of wire hanging of mice after TBI. In addition, *β*-carotene could maintain the BBB permeability as indicated by the EB extravasation and ameliorate oxidative stress as showed by the increased SOD level and decreased MDA levels. The mechanistic study demonstrated that *β*-carotene activated the Keap1-Nrf2 signaling pathway and promoted the expression of HO-1 and NQO1 [169]. Therefore, *β*-carotene provides a neuroprotective effect in TBI via inhibiting oxidative stress through the Nrf2 pathway.

### 5.18. Astaxanthin

Astaxanthin is a carotenoid and is commonly found in certain plants and animals, such as salmon, rainbow trout, shrimp, and lobster [170]. Emerging evidence indicates that astaxanthin exhibits multiple biological activities, including antiageing, anticancer, heart protection, and neuroprotection [171]. Recently, astaxanthin was reported to present neuroprotection in CCI-induced TBI mice, such as increasing NSS score and immobility time and increasing rotarod time and latency to immobility. In addition, astaxanthin increased SOD1 levels and inhibited the protein expression of cleaved caspase 3 and the number of TUNEL-positive cells, suggesting that astaxanthin exerted antioxidative and antiapoptotic effects. The mechanistic study demonstrated that astaxanthin increased the protein and mRNA expressions of Nrf2, HO-1, NQO1, and SOD1 [172]. Moreover, in weight drop-induced TBI mice, astaxanthin significantly reduced brain edema and improved behavioral functions including neurological scores, rotarod performance, beam walking performance, and falling latency during the hanging test. In addition, astaxanthin improved neuronal survival indicated by Nissl staining. Furthermore, astaxanthin exerted an antioxidative effect by increasing the SOD1 protein expression and inhibited neuronal apoptosis by reducing the level of cleaved caspase 3 and the number of TUNEL-positive cells. The mechanistic study revealed that astaxanthin promoted the activation of the Nrf2 signaling pathway as demonstrated by the increased mRNA levels and protein expressions of Nrf2, HO-1, and NQO1, while the inhibition of Prx2 or SIRT1 reversed the antioxidative and antiapoptotic effect of astaxanthin. Therefore, astaxanthin activated the SIRT1/Nrf2/Prx2/ASK1 signaling pathway in TBI. Moreover, astaxanthin also provided a neuroprotective effect in H_2_O_2_-induced primary cortical neurons by reducing oxidative damage and inhibiting apoptosis via the SIRT1/Nrf2/Prx2/ASK1/p38 signaling pathway [173]. Therefore, astaxanthin exerts a neuroprotective effect including antioxidation and antiapoptosis via activating the Nrf2 signaling pathway in TBI.

### 5.19. Lutein

Lutein, a natural carotenoid, is commonly found in a variety of flowers, vegetables, and fruits, such as *Calendula officinalis*, spinach, and *Brassica oleracea* [174]. Accumulating studies demonstrate that lutein is a potent antioxidant and exhibits benefits in various diseases, including ischemia/reperfusion injury, diabetic retinopathy, heart disease, AD, and TBI [175]. In severe TBI rats, the administration of lutein significantly increased the inhibition of skilled motor function and reversed the increase in contusion volume of TBI rats. In addition, lutein suppressed the inflammatory response by decreasing the levels of TNF-*α*, IL-1*β*, IL-6, and Monocyte chemoattractant protein-1 (MCP-1). Meanwhile, lutein decreased ROS production and increased SOD and GSH activity, suggesting that lutein attenuated TBI-induced oxidative damage. Moreover, the mechanistic study found that lutein inhibited the protein expression of intercellular adhesion molecule-1 (ICAM-1), COX-2, and NF-*κ*B, while increasing the protein expression of ET-1 and Nrf2. Therefore, the neuroprotective effect of lutein in TBI may be regulated via the NF-*κ*B/ICAM-1/Nrf2 signaling pathway [176]. It is known that zeaxanthin and lutein are isomers and have identical chemical formulas. Recently, it is reported that lutein/zeaxanthin exerted a neuroprotective effect in TBI mice induced by a liquid nitrogen-cooled copper probe, and the brain infarct and brain swelling were remarkably declined by lutein/zeaxanthin. The protein expression of Growth-Associated Protein 43 (GAP43), ICAM, neural cell adhesion molecule (NCAM), brain-derived neurotrophic factor (BDNF), and Nrf2 were increased, while the protein expression of GFAP, IL-1*β*, IL-6, and NF-*κ*B was inhibited by lutein/zeaxanthin [177]. Therefore, lutein/zeaxanthin presents antioxidative and anti-inflammatory effects via the Nrf2 and NF-*κ*B signaling pathways.

### 5.20. Sodium Aescinate

Sodium aescinate (SA) is a mixture of triterpene saponins isolated from the seeds of *Aesculus chinensis* Bunge and chestnut [178]. Amounting studies show that SA exerts anti-inflammatory, anticancer, and antioxidative effects [179–181]. In addition, SA has been reported to exhibit neuroprotective effect in 1-methyl-4-phenyl-1,2,3,6-tetrahydropyridine (MPTP)-induced PD mice and mutant huntingtin (mHTT) overexpressing HT22 cells [182, 183]. A recent study reported that SA could attenuate brain injury in weight drop-induced TBI mice [182]. The intraperitoneal administration of SA significantly decreased NSS, brain water content, and lesion volume of mice after TBI. A further study found that SA suppressed TBI-induced oxidative stress as evidenced by the decreased MDA levels and increased GPx activity. The Nissl staining images displayed that SA increased the viability of neurons, and the TUNEL staining showed that SA inhibited neuronal apoptosis. Meanwhile, SA decreased the ratio of Bax/Bcl-2 and the cleaved form of caspase-3, while increasing the release of cytochrome c from mitochondria into the cytoplasm. The mechanistic study demonstrated that SA promoted the translocation of Nrf2 from the cytoplasm into the nuclear and subsequently increased the expression of HO-1 and NQO1. Moreover, the neuroprotective effect and mechanism of action of SA have been confirmed in scratch injury-induced TBI primary neurons and Nrf2-KO mice after TBI. Therefore, SA exerts a neuroprotective effect in TBI via activating the Nrf2 signaling pathway.

### 5.21. Melatonin

Melatonin, commonly found in plants, animals, fungi, and bacteria, plays an important role in the regulation of the biological clock [184]. Melatonin as a dietary supplement is widely used to treat insomnia. Emerging evidence indicates that melatonin exerts neuroprotection in various diseases including brain injury, spinal cord injury, and cerebral ischemia [185]. In addition, melatonin is demonstrated to be a potent antioxidant with the ability to reduce oxidative stress, inhibit the inflammatory response, and attenuate neuronal apoptosis [186]. In craniocerebral trauma, melatonin showed a neuroprotective effect due to its antioxidative, anti-inflammatory, and inhibitory effects on activation adhesion molecules [187]. In Marmarou's weight drop-induced TBI mice, melatonin significantly inhibited neuronal degeneration and reduced cerebral edema in the brain. Meanwhile, melatonin also attenuated the oxidative stress induced by TBI as evidenced by the decreased MDA levels and 3-NE expression, as well as increased GPx and SOD levels. The mechanistic study demonstrated that melatonin increased the nuclear translocation of Nrf2 and promoted the protein expression and mRNA levels of HO-1 and NQO1, while the KO of Nrf2 could partly reverse the neuroprotective effect of melatonin, including antioxidation, inhibition of neuronal degeneration, and alleviation of cerebral edema in mice after TBI. Therefore, melatonin provides a neuroprotective effect in TBI via the Nrf-ARE signaling pathway [188]. Due to the complex pathophysiology of TBI, the combinational use of melatonin and minocycline, a bacteriostatic agent reported to inhibit neuroinflammation, did not exhibit a better neuroprotective effect than either agent alone. The dosing and/or administration issues may attribute to this result [189]. Therefore, the optimal combination should be explored for the treatment of TBI.

### 5.22. Sinomenine

Sinomenine is an alkaloid compound that is isolated from the roots of climbing plants including *Sinomenium acutum* (Thunb.) Rehd. et Wils. and *Sinomenium acutum* var. cinereum Rehd. et Wils [190]. Sinomenine has been demonstrated to exhibit an antihypertensive and anti-inflammatory effect and is commonly used to treat various acute and chronic arthritis, rheumatism, and rheumatoid arthritis (RA). In addition, sinomenine provides a neuroprotective effect in Marmarou's weight drop-induced TBI mice. The administration of sinomenine significantly increased the grip test score and decreased brain water content. In addition, the neuronal viability was increased by sinomenine as shown by the increased NeuN-positive neurons and decreased TUNEL-positive neurons. Meanwhile, sinomenine increased Bcl-2 protein expression and decreased cleaved caspase-3 expression. Furthermore, sinomenine attenuated oxidative stress by decreasing MDA levels and increasing SOD and GPx activity. The mechanistic study revealed that sinomenine promoted the nuclear translocation of Nrf2 and increased the mRNA and protein expression of HO-1 and NQO1 in mice after TBI [191]. Therefore, sinomenine, acting as a potent anti-inflammatory agent, provides antiapoptotic and antioxidative effects in TBI via the Nrf2-ARE signaling pathway.

### 5.23. Sulforaphane

Sulforaphane, also known as isothiocyanate, is commonly found in certain kinds of vegetables, including cabbage, broccoli, and cauliflower [192]. Emerging evidence indicates that sulforaphane is widely used to treat prostate cancer, autism, asthma, and many other diseases [193–195]. In addition, sulforaphane also showed a neuroprotective effect in TBI. For example, sulforaphane decreased BBB permeability in CCI-induced TBI rats as evidenced by the decreased EB extravasation and the relative fluorescence intensity of fluorescein [196]. Meanwhile, the loss of tight junction proteins (TJs) including occluding and claudin-5 was attenuated by sulforaphane. The mechanistic study found that sulforaphane increased the mRNA level of Nrf2-driven genes including GST-alpha3(GST*α*3), GPx, and HO-1, as well as enhanced the enzymatic activity of NQO1 in the brain and brain microvessels of TBI mice, suggesting that sulforaphane activated the Nrf2-ARE signaling pathway to protect BBB integrity. Furthermore, sulforaphane could reduce brain edema as evidenced by the decrease in brain water content, which was closely associated with the attenuation of AQP4 loss in the injury core and the further increase of AQP4 level in the penumbra region [197]. Moreover, the Morris water maze (MWZ) test showed that sulforaphane improved spatial memory and spatial working memory. Meanwhile, TBI-induced oxidative damage was significantly attenuated by sulforaphane as demonstrated by the reduced 4-HNE levels [198]. In addition, sulforaphane also attenuated 4-HNE induced dysfunction in isolated cortical mitochondria [158]. Taken together, sulforaphane provides a neuroprotective effect in TBI via the activation of the Nrf2-ARE signaling pathway.

## 6. Conclusions and Perspective

**Figure 4:**
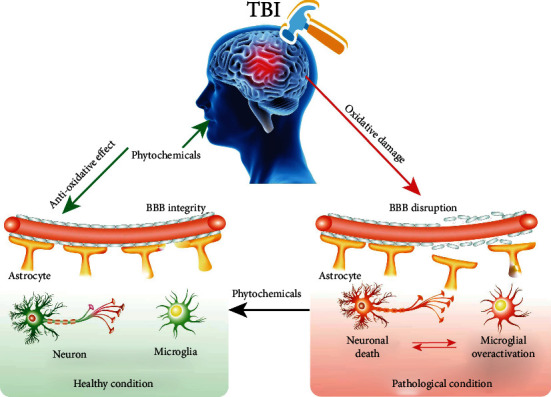
The potential therapy of phytochemicals for TBI. Oxidative damage in TBI plays an important role in the pathology of TBI, including BBB disruption and then neuronal death and microglial overactivation, while the treatment of phytochemicals with antioxidative properties can improve BBB integrity and then recover neuronal viability and inhibit microglial overactivation.

It is known to us that TBI causes irreversible primary mechanical damage, followed by secondary injury. Studies have shown that multiple mechanisms contribute to the development of TBI during secondary injury, mainly including inflammatory response, oxidative stress, mitochondrial dysfunction, BBB disruption, and otherwise. Among them, oxidative stress leads to mitochondrial dysfunction, BBB disruption, and neuroinflammation. Therefore, oxidative stress plays a central role in the pathogenesis of TBI. Nrf2 is a conserved bZIP transcription factor, and the activation of the Nrf2 signaling pathway protects against oxidative damage. Under stress conditions or the treatment of Nrf2 activators, Nrf2 is translocated from the cytoplasm into the nucleus where it protects against oxidative damage via the ARE-mediated transcriptional activation of genes, including HO-1, NQO1, and GCLC, thereby inhibiting mitochondrial dysfunction, apoptosis, inflammation, and oxidative damage. Therefore, targeting the activation of the Nrf2 signaling pathway is a promising therapeutical strategy for TBI. To date, increased Nrf2 activators were reported to exert neuroprotective effects in various neurodegenerative diseases, cerebral ischemia, cerebral hemorrhage, and TBI [199, 200]. Phytochemicals are rich and isolated from fruits, vegetables, grains, and other medicinal herbs. In this review, polyphenols, terpenoids, natural pigments, and other phytochemicals were summarized. They exhibit potent neuroprotective effects, including the improvement of BBB integrity, recovery of neuronal viability, and inhibition of microglial overactivation via the Nrf2-mediated oxidative stress response (Figure 4).

Although a large number of studies have demonstrated the neuroprotective effect of most of the phytochemicals in vivo and in vitro models of TBI, there is a lack of effective clinical application evidence. In addition, little is known about the safety and pharmacokinetics of these phytochemicals. Therefore, increasing studies are needed to be performed to accelerate the process of phytochemicals entering the clinic. In the later period of TBI recovery, the selective permeability of BBB also gradually recovered. At this time, BBB is a big obstacle, which greatly limits the neuroprotective effect of drugs, and the use of drugs based on nanomaterials effectively improves the BBB permeability of drugs, bringing new hope for these phytochemicals. In addition, the combinational use of phytochemicals targeting multi-targets such as Nrf2, NF-*κ*B, NADPH oxidase-2 (NOX-2) with gene, and stem cell therapy will be a promising strategy for the treatment of TBI.

## Figures and Tables

**Table 1:** Phytochemicals from various plants possess multiple pharmacological effects via the antioxidant mechanism in various in vitro and in vivo models of TBI.

Phytochemicals	Plants	Models	Pharmacological effects	Detected markers	Antioxidant mechanism	Ref
Polyphenol						
Quercetin	Onions, tomatoes, etc.	Weight drop-induced TBI mice/rats,	Improved behavioral function, neuronal viability, and mitochondrial function; reduced brain edema and microgliosis, oxidative damage and nitrosative stress, neuronal apoptosis, inflammatory response	Motor coordination; latency period; NSS; brain water content; MDA; SOD; catalase; GPx; lipid peroxidation; neuronal morphology; cytochrome c; Bax; MMP; ATP; Iba-1; TNF-*α*; iNOS; cNOS; IL-1*β*; Nrf2; HO-1	Nrf2 pathway	[74–77]
Curcumin	*Curcuma longa*	FPI-induced TBI rats; Feeney or weight drop-induced TBI WT, Nrf2-KO or TLR4-KO mice; LPS-induced microglia or the co-culture of neuron and microglia	Improved cognitive function; reduced axonal injury, neuronal apoptosis, inflammatory response, and oxidative damage	NSS; brain water content; Tuj1; H&E; Nissl; Congo red, silver, TUNEL, MPO, and FJC staining; caspase 3; Bcl-2; NeuN/BrdU double labeling; Iba-1; GFAP; TNF-*α*; IL-6; IL-1*β*; MCP-1; RANTES; CD11B; DCX; TLR4; MyD88; NF-*κ*B; IkB; AQP4; Nrf2; HO-1; NQO1; PERK; eIF2*α*; ATF4; CHOP; GSK3*β*; p-tau; *β*-APP; NF-H	Nrf2 pathway; PERK/Nrf2 pathway	[80, 81, 86–88]
Formononetin	Red clover	Weight drop-induced TBI rats	Reduced brain edema, pathological lesions, inflammatory response, and oxidative damage,	NSS; brain water content; H&E and Nissl staining; neuronal ultrastructural organization; SOD; GPx; MDA; TNF-*α*; IL-6; COX-2; IL-10; Nrf2	Nrf2 pathway	[91, 95]
Baicalin	*Scutellaria baicalensis*	Weight drop-induced TBI rats	Improved behavioral function and neuronal survival; reduced brain edema, oxidative damage, BBB disruption, and mitochondrial apoptosis	NSS; brain water content; EB leakage, Nissl, and TUNEL staining, grip test score; cleaved caspase 3; Bcl-2, cytochrome c, p53, SOD, MDA, GPx, NeuN, Nrf2, HO-1, NQO1, AMPK, mTOR, LC3, Beclin-1, p62	Akt/Nrf2 pathway	[96, 97]
Catechin	Cocoa, tea, grapes, etc.	CCI- or weight drop-induced TBI rats	Improved long-term neurological outcomes, neuronal survival, and white matter recovery; reduced brain edema, brain lesion volume, neurodegeneration, inflammatory response, BBB disruption, neutrophil infiltration, and oxidative damage	NSS; brain water content; brain infarct volume; forelimb score; Hindlimb score; latency; quadrant time; EB extravasation; ZO-1; Occludin; TNF-*α*；IL-1*β*；IL-6; iNOS; arginase; TUNEL, PI, FJB, Cresyl violet, and MPO staining; myelin; caspase 3; caspase 8; Bcl-2; Bax; BDNF; ROS; MMP-2; MMP-9; Nrf2, Keap1, SOD1; HO-1; NQO1; NF-*κ*B	Nrf2-dependent and Nrf2-independent pathways	[103, 104]
Fisetin	*Cotinus coggygria*, onions, cucumbers, etc.	Weight drop-induced TBI mice	Improved neurological function; reduced cerebral edema, brain lesion, oxidative damage, and BBB disruption	NSS; brain water content; grip score; EB extravasation; lesion volume; MDA; GPx Nissl and TUNEL staining; caspase 3; Bcl-2; Bax; Nrf2, HO-1; NQO1; TLR4; NF-*κ*B; NeuN; TNF-*α*；IL-1*β*；IL-6; MPP-9; ZO-1; EB leakage	Nrf2-ARE signaling pathway	[109, 110]
Luteolin	Carrots, green tea, celery, etc.	Marmarou's weight drop-induced TBI mice/rats; scratch injury-induced TBI primary neurons	Improved motor performance, and learning and memory; reduced cerebral edema, apoptosis index, and oxidative damage	Latency time; brain water content; grip score; MDA; GPx; catalase; SOD; TUNEL, H&E, Cresyl violet, and TB staining; ROS; LDH release assay; Nrf2; HO-1; NQO1	Nrf2-ARE signaling pathway	[116, 117]
Isoliquiritigenin	*Sinofranchetia chinensis*, *Glycyrrhiza uralensis*, and *Dalbergia odorifera*	CCI-induced TBI mice/rats; ODG-induced SH-SY5Y cells	Improved motor performance, cognitive function, and cell viability; reduced cerebral edema, neuronal apoptosis, inflammatory response, BBB damage, and oxidative damage	Garcia neuroscore; MWM test; beam-balance latency; beam-walk latency; brain water content; contusion volume; EB extravasation; apoptosis rate; MDA; GPx; SOD; H_2_O_2_; H&E and Nissl staining; GFAP; NFL; AQP4; caspase 3; Bcl-2; Bcl-xL; Bax; Nrf2, HO-1; NQO1; TNF-*α*; INF-*γ*; IL-1*β*; IL-6; IL-10; Iba-1; CD68; AKT; GSK3*β*; P-120; Occludin; NF-*κ*B; IkB; CCK-8 assay	Nrf2-ARE signaling pathway	[121–123]
Tannic acid	Green and black tea, nuts, fruits, and vegetables	CCI-induced TBI mice/rats; ODG-induced SH-SY5Y cells	Improved behavioral performance; reduced cerebral edema, neuronal apoptosis, inflammatory response, and oxidative damage	Grip test score; Rotarod test; beam balance; brain water content; GSH; LPO; GST; GPx; CAT; SOD; Nissl staining; caspase 3; Bcl-2; Bax; PARP; Nrf2; PGC-1*α*; Tfam; HO-1; NQO1; TNF-*α*; IL-1*β*; 4HNE; GFAP	PGC-1*α*/Nrf2/HO-1 pathway	[133]
Ellagic acid	Various berries, walnuts, and nuts	Experimental diffuse TBI rats; CCl4-induced brain injury rats	Improved memory, hippocampus electrophysiology and long-term potentiation deficit; reduced neuronal apoptosis, inflammatory response, oxidative damage, and BBB disruption	Initial latency; step through latency; EB leakage; NSS; MDA; GSH; CAT; caspase 3; Bcl-2; NF-*κ*B; PARP; Nrf2; Cox-2, VEGF; TNF-*α*； IL-1*β*; IL-6	Nrf2 signaling pathway	[134, 135]
Breviscapine	Erigeron	Weight drop- or CCI-induced TBI rats	Improved neurobehavior; reduced neuronal apoptosis, inflammatory response, and oxidative damage	NSS; TUNEL staining; MDA; GSH; CAT; caspase 3; Bcl-2; Bax; IL-6; Nrf2; HO-1; NQO1; GSK3*β*; SYP	Nrf2 signaling pathway	[138–140]
Terpenoids						
Asiatic acid	*Centella asiatica*	CCI-induced TBI rats	Improved neurological deficits; inhibited brain edema, neuronal apoptosis, and oxidative damage	NSS; brain water content; TUNEL staining; MDA; 4-HNE; 8-OhdG; Nrf2; HO-1	Nrf2 signaling pathway	[149]
Aucubin	*Eucommia ulmoides*	Weight drop-induced TBI mice; H_2_O_2_-induced primary cortical neurons	Improved neurological deficits, and cognitive function; reduced brain edema, neuronal apoptosis and loss, inflammatory response, and oxidative damage	NSS; brain water content; TUNEL and Nissl staining; MWM test; Bcl-2; Bax; CC3; MAP2; MMP-9; MDA; SOD; GSH; GPx; 8-HdG; NeuN; Iba-1; HMGB1; TLR4; MyD88; NF-*κ*B; iNOS; COX2; IL-1*β*; Nrf2; HO-1; NQO1	Nrf2 signaling pathway	[155]
Ursolic acid	Apples, bilberries, lavender, hawthorn, etc.	Weight drop-induced TBI mice	Improved neurobehavioral and mitochondrial function; reduced brain edema, oxidative damage, and neuronal cytoskeletal degradation	NSS; brain water content; TUNEL and Nissl staining; MDA; SOD; GPx; AKT; 4-HNE; 3-NE; ADP rate; succinate rate; Spectrin; Nrf2; HO-1; NQO1	AKT/Nrf2 signaling pathway	[158]
Carnosic acid	*Rosmarinus officinalis* and *Salvia officinalis*.	CCI-induced acute post-TBI mice;	Improved motor and cognitive function, and neuronal viability; reduced brain edema, neuronal apoptosis and loss, inflammatory response, and oxidative damage	Duration of apnea; mitochondrial respiration; Barnes maze test; novel object recognition (NOR) task; GFAP; Iba-1; NeuN; MAP2; vGlut1; HO-1	Nrf2-ARE signaling pathway	[157–159]
Natural pigments						
Fucoxanthin	*Rosmarinus officinalis* and *Salvia officinalis*	Weight drop-induced TBI mice; scratch injury-induced TBI primary cortical neurons	Improved neurobehavioral function, and neuronal viability; reduced brain edema, neuronal apoptosis, and oxidative damage	NSS; grip test score; brain water content; lesion volume; TUNEL staining; caspase 3; PARP; cytochrome c; MDA; GPx; ROS; LC3; NeuN; p62; Nrf2; HO-1; NQO1	Nrf2-ARE and Nrf2-autophagy	[170]
*β*-Carotene	Fungi, plants, and fruits	Weight drop-induced TBI mice	Improved neurological function; reduced brain edema, BBB disruption, neuronal apoptosis, and oxidative damage	Neurological deficit score; wire hanging; brain water content; EB extravasation; MDA; SOD; NeuN; Nissl and TUNEL staining; caspase 3; Bcl-2; Keap1; Nrf2; HO-1; NQO1	Keap1-Nrf2 signaling pathway	[174]
Astaxanthin	Salmon, rainbow trout, shrimp, and lobster	CCI- or weight drop-induced TBI mice; H_2_O_2_-induced primary cortical neurons	Improved neurological, motor, and cognitive function; reduced brain edema, BBB disruption, neuronal apoptosis, and oxidative damage	NSS; Rotarod test time; neurological deficit scores; rotarod performance; beam walking score; wire hanging test; MWM test; brain water content; 8-OhdG; immobility time; latency to immobility; SOD1; MDA; H_2_O_2_; GSH; ROS; CC3; Nissl, Cresyl violet, and TUNEL staining; Prx2; SIRT1; ASK1; p38; NeuN; Bax; Bcl-2; caspase 3; Nrf2; HO-1; NQO1	Nrf2 signaling pathway; SIRT1/Nrf2/Prx2/ASK1/p38 signaling pathway	[172, 173]
Lutein	*Calendula officinalis*, spinach, and *Brassica oleracea*	CCI-induced STBI mice; H_2_O_2_-induced primary cortical neurons	Improved motor and cognitive function; reduced brain edema, contusion volume, inflammatory response; and oxidative damage	Forelimb reaching test; immobility time; latency to immobility; brain water content; 8-OhdG; TNF-*α*; IL-1*β*; IL-6; MCP-1; ROS; SOD; GSH; ICAM-1; COX-2 NF-*κ*B; ET-1; MDA; H_2_O_2_; CC3; Nissl, Cresyl violet, and TUNEL staining; Prx2; SIRT1; ASK1; p38; NeuN; Bax; Bcl-2; caspase 3; Nrf2; HO-1; NQO1	ICAM-1/Nrf-2 signaling pathway	[176, 177]
Others						
Sodium aescinate	*Aesculus chinensis* Bunge and chestnut	Weight drop-induced TBI mice; scratch injury-induced primary cortical neurons	Improved neurological function; reduced brain edema, inflammatory response; and oxidative damage	NSS; brain water content; lesion volume; MDA; GPx; Nissl and TUNEL staining; Bax; Bcl-2; cytochrome c; caspase 3; cell survival; ROS; Nrf2; HO-1; NQO1	Nrf2-ARE pathway	[187]
Melatonin	Plants, animals, fungus, and bacteria	Marmarou's weight drop-induced TBI mice	Reduced brain edema, neuronal degeneration and apoptosis, and oxidative damage	Brain water content; MDA; 3-NT; GPx; SOD; FJC staining; NeuN; Beclin-1; Nrf2; HO-1; NQO1	Nrf2-ARE pathway	[193]
Sinomenine	*Sinomenium acutum* (Thunb.) Rehd. Et Wils. and *Sinomenium acutum* var. cinereum Rehd. Et Wils.	Marmarou's weight drop-induced TBI mice	Improved motor performance; reduced brain edema, neuronal apoptosis, and oxidative damage	Grip test score; brain water content; NeuN and TUNEL staining; Bcl-2; caspase 3; MDA; GPx; SOD; Nrf2; HO-1; NQO1	Nrf2-ARE pathway	[196]
Sulforaphane	Vegetable, including cabbage, broccoli, and cauliflower	CCI-induced TBI mice	Improved motor performance and cognitive function, reduced brain edema, BBB permeability, mitochondrial dysfunction, and oxidative damage	MWZ test; EB extravasation; brain water content; Occludin; Claudin-5; RECA-1; vWF; EBA; ZO-1; AQP4; GPx; GST*α*3; 4-HNE; ADP rate; succinate rate; NeuN; Nrf2; HO-1; NQO1	Nrf2 signaling pathway	[158, 196–198]

## Data Availability

The data used to support the findings of this study are available from the corresponding author upon request.
